# Gender-Related Effectiveness of Personalized Post-COVID-19 Rehabilitation

**DOI:** 10.3390/jcm13040938

**Published:** 2024-02-06

**Authors:** Alicja Rzepka-Cholasińska, Jakub Ratajczak, Piotr Michalski, Michał Kasprzak, Agata Kosobucka-Ozdoba, Łukasz Pietrzykowski, Klaudyna Grzelakowska, Jacek Kubica, Jacek Kryś, Aldona Kubica

**Affiliations:** 1 Department of Cardiac Rehabilitation and Health Promotion, Nicolaus Copernicus University in Torun, Collegium Medicum in Bydgoszcz, 85-094 Bydgoszcz, Poland; jakub.ratajczak@cm.umk.pl (J.R.); piotr.michalski@cm.umk.pl (P.M.); a.kosobucka@cm.umk.pl (A.K.-O.); lukasz.pietrzykowski@cm.umk.pl (Ł.P.); aldona.kubica@gmail.com (A.K.); 2 Department of Cardiology and Internal Medicine, Nicolaus Copernicus University in Torun, Collegium Medicum in Bydgoszcz, 85-094 Bydgoszcz, Poland; michal.kasprzak@cm.umk.pl (M.K.); k.grzelakowska@doktorant.umk.pl (K.G.); jkubica@cm.umk.pl (J.K.); 3 Department of Health Economics, Nicolaus Copernicus University in Torun, Collegium Medicum in Bydgoszcz, 85-094 Bydgoszcz, Poland; jacek.krys@cm.umk.pl

**Keywords:** fatigue, physical activity, post-COVID-19 syndrome, bioimpedance, body composition, hand grip, rehabilitation

## Abstract

**Background:** Post-COVID-19 syndrome (PCS) may affect a substantial proportion of patients who have had COVID-19. The rehabilitation program might improve the physical capacity, functioning of the cardiopulmonary system, and mental conditions of these patients. This study aimed to investigate the effectiveness of personalized rehabilitation in patients with PCS according to gender. **Methods**: Adults who underwent a 6-week personalized PCS rehabilitation program were enrolled in a prospective post-COVID-19 Rehabilitation (PCR-SIRIO 8) study. The initial visit and the final visit included the hand-grip strength test, the bioimpedance analysis of body composition, and the following scales: modified Borg’s scale, Modified Fatigue Impact Scale (MFIS), Functioning in Chronic Illness Scale (FCIS), modified Medical Research Council (mMRC) dyspnea scale, and tests: 30 s chair stand test (30 CST), Six-Minute Walk Test (6MWT), Short Physical Performance Battery test (SPPB)e. **Results**: A total of 90 patients (54% female) underwent the rehabilitation program. Rehabilitation was associated with an increase in skeletal muscle mass (24.11 kg vs. 24.37 kg, *p* = 0.001) and phase angle (4.89° vs. 5.01°, *p* = 0.001) and with a reduction in abdominal fat tissue volume (3.03 L vs. 2.85 L, *p* = 0.01), waist circumference (0.96 m vs. 0.95 m, *p* = 0.001), and hydration level (83.54% vs. 82.72%, *p* = 0.001). A decrease in fat tissue volume and an increase in skeletal muscle mass were observed only in females, while an increase in grip strength was noticed selectively in males. Patients’ fatigue (modified Borg’s scale, MFIS), physical capacity (30 CST, 6MWT), balance (SPPB), dyspnea (mMRC), and functioning (FICS) were significantly improved after the rehabilitation regardless of gender. **Conclusions**: Personalized rehabilitation improved the body composition, muscle strength, and functioning of patients diagnosed with PCS. The beneficial effect of rehabilitation on body composition, hydration, and phase angle was observed regardless of gender.

## 1. Introduction

The long-term consequences of coronavirus disease 2019 (COVID-19) are varied and not fully known. The occurrence of long-term symptoms within three months from the onset of COVID-19 that last for at least two months was defined as post-COVID-19 syndrome (PCS) [1]. It is estimated that PCS may affect up to 80% of patients after SARS-CoV-2 infection [1]. Patients with PCS, despite somatic symptoms, are also affected by mental and cognitive disorders, chronic stress, and decreased quality of life [1,2]. Treatment of patients with PCS should be complemented by complex and personalized rehabilitation [2]. Previous studies showed that women were more symptomatic and more likely to have prolonged symptoms after COVID-19 infection [3,4,5]. Gender-related differences were also found regarding specific types of rehabilitation. Women who underwent a cardiac rehabilitation program had a lower baseline physical capacity measured by peak oxygen uptake and might have a lower training response when undergoing cardiac rehabilitation [6]. However, recent studies indicated that high-intensity interval training combined with resistance training of the lower limbs might be effective [7,8]. According to the available data, as many as three to four times more women participate in rehabilitation due to PCS [9]. This study aimed to investigate the effectiveness of the program of personalized rehabilitation in patients diagnosed with PCS according to gender.

## 2. Materials and Methods

Adults (≥18 years of age) who had at least one confirmed polymerase chain reaction (PCR) COVID-19 infection and received general practitioner referral for the PCS rehabilitation program could be included in the prospective Post-COVID-19 Rehabilitation SIRIO 8 (PCR-SIRIO 8) study. Patients were qualified if the post-COVID-19 dyspnea persisted for at least 3 months. The COVID-19 diagnosis had to be documented in the patient’s medical file. The period between the infection and inclusion into this study could not exceed one year. The original program of personalized rehabilitation was conducted at the Department of Cardiac Rehabilitation and Health Promotion at the Antoni Jurasz University Hospital No. 1 in Bydgoszcz. The exclusion criteria used were as follows: no clinically relevant dyspnea defined as grade 0 in the modified Medical Research Council (mMRC) dyspnea scale; a status of an incapacitated person; cognitive disorders or psychological state preventing informed consent; medical contraindications to participate in the rehabilitation program (musculoskeletal dysfunction and other comorbidities); inability to perform bioelectrical impedance measurement due to metal medical implants. Only patients who underwent the complete rehabilitation program were included in the final analysis of the PCR-SIRIO 8 study.

**Figure 1:**
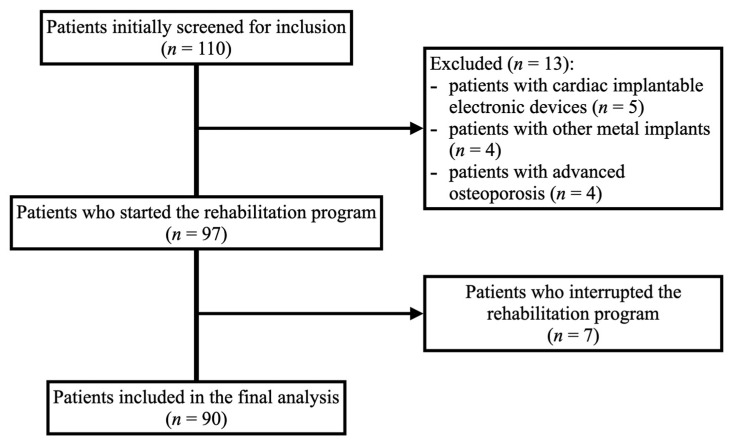
Post-COVID-19 Rehabilitation (PCR-SIRIO 8) study—number of patients initially screened, included, and excluded for further analysis.

A total of 110 patients were assessed under the inclusion criteria, of which 97 started the rehabilitation and 90 were included in the final analysis (49 women and 41 men). Figure 1 shows detailed information regarding the inclusion process.

A cardiologist consulted all participants before starting the rehabilitation program to exclude possible contraindications to physical exercise. All patients were also assessed by the physiotherapist during the first and last visit. The rehabilitation was performed on an outpatient basis for a six-week period. All patients in the study were exercising according to the high-intensity interval training protocol. It consisted of three parts: a warm-up (5 min), a proper part (20 min), and a calming phase (5 min). The warm-up phase consisted of breathing and active exercises. The proper phase included the following: active exercises using a gymnastic stick and small rehabilitation balls, balance training with a sensorimotor cushion disk, active exercises with resistance using b™ resistance band and weights (0.5–4 kg), aerobic exercises with 25 cm step, and respiratory exercises with resistance using TheraBand™ (Akron, OH, USA) resistance band and water bottle. The calming phase included stretching and breathing exercises. The training sessions were performed three times per week, except resistance exercises, which were performed once a week in the initial two weeks, twice a week in the following two weeks, and three times in the final two weeks of the rehabilitation program. All training sessions were supervised by a qualified and experienced physiotherapist to ensure adequate adherence to the exercise regimen. The physiotherapist at the initial visit qualified the patient for rehabilitation based on the initial activity level, muscle strength assessed with the Medical Research Council (MRC) Scale for Muscle Strength, and severity of dyspnea based on the mMRC scale for one of the following groups:

- Patients with low initial activity levels and low muscle strength (MRC < Grade 3) or with severe dyspnea (mMRC ≥ 3) performed resistance exercises with lower weights (0.5 kg). Active exercises in this group were performed in a sitting position, and each exercise requiring higher intensity was interspersed with breathing exercises. The duration of a training session was up to 30 min. The rehabilitation began with 1 series with 10 repetitions of each exercise in the initial week and progressed to 3 sessions with 20 repetitions in the final week. Training intensity was based on the heart rate reserve and aimed at 30% in the initial week, and up to 60% in the final week.
- Patients with low to medium initial activity levels and medium muscle strength (MRC = Grade 3) or with medium dyspnea (mMRC = 2) performed resistance exercises with heavier and increasing weights, starting with 1 kg in the initial week and 1.5 kg in the final week. In this group, active exercises were performed in a standing position and breathing exercises in a sitting position. The duration of a single session was up to 45 min. The rehabilitation began with 1 series with 12 repetitions of each exercise in the initial week and increased to 3 sessions with 25 repetitions in the final week. Training intensity was based on the heart rate reserve and aimed at 40% in the initial week, and up to 70% in the final week.
- Patients with higher initial activity levels and muscle strength (MRC > 4) or with mild dyspnea (mMRC = 1) performed resistance exercises with heavier and increasing weights, starting with 2 kg in the initial week and 4 kg in the final week. In this group, active exercises were performed in a standing position, and breathing exercises both in a sitting and standing position, and the duration of a single session was up to 60 min. The rehabilitation started with 1 series with 15 repetitions of each exercise in the initial week and increased to 3 sessions with 30 repetitions in the final week. Training intensity was based on the heart rate reserve and aimed at 50% in the initial week, and up to 80% in the final week.

The initial assessment also included patients’ comorbidities and functional status (balance, gait, muscle strength). Based on the above-mentioned criteria, individual short- and long-term goals were defined. The first was to be achieved by the end of the 6-week rehabilitation program, and the latter after completing the rehabilitation program. The leading physiotherapist individualized the rehabilitation program for each patient by adapting the time of the physical training, loads, and type of exercises according to the observed progress. The rehabilitation was supplemented by therapeutic education, including methods of dealing with PCS, learning how to breathe, cough, and expectorate properly (techniques improving respiratory system functioning), authigenic training, and properly performing physical activities. The effectiveness of the rehabilitation program was assessed by comparing the differences between two time points, i.e., during the initial visit and the final visit after six weeks. In the analysis, the following parameters were assessed:

1. The grip strength of both hands tested using a Baseline Pneumatic Squeeze Bulb Dynamometer 30 PSI (Fabrication Enterprises Inc., White Plains, NY, USA). The measurement was performed three times at every visit and the best attempt was used for the analysis. The measurement was made in the sitting position with the elbow resting at an angle of 90 degrees [10];
2. Bioelectrical impedance analysis with the assessment of skeletal muscle mass, muscle mass of the left and right extremities, lean mass, abdominal fat volume, phase angle (PhA, an indicator of the functional state of cell membranes; the higher the value, the better the cell health), and the hydration rate [10,11]. The body composition analysis was performed using a calibrated digital flat platform scale SECA mBCA 515 (Seca GmbH & Co. KG, Hamburg, Germany) measuring with the accuracy of 0.1 kg [11,12], considering height, waist circumference, gender, age, and race. To perform the measurement, the patient stood with the legs slightly apart, placing bare feet and hands on designated areas of the SECA mBCA 515 scale. The patient remained motionless for 17 s.
3. Tests and scales assessing functional and psychological state:
  - mMRC dyspnea scale (grades 0–4);
  - Modified Fatigue Impact Scale (MFIS, impact of fatigue on patient’s daily life, 0–84 points);
  - Modified Borg scale (the assessment of perceived fatigue and dyspnea, score 0–10);
  - 30 s chair stand test (30 CST, the assessment depending on gender and age groups);
  - Short Physical Performance Battery test (SPPB, the assessment of balance and walking speed over 3 and 4 m distances, 0–12 points);
  - Six-Minute Walk Test (6MWT, the assessment of physical fitness);
  - Functioning in Chronic Illness Scale (FCIS, subscales FCIS 1 available 8–40 points, FCIS 2 available 8–40 points, and FCIS part 3 available 8–40 points; total FCIS score 24–120 points) [2,5,13]. The FCIS allows for the diagnosis of deficit areas in patients and the implementation of appropriate interventions. The FCIS 1 subscale mainly refers to the patient’s physical efficiency, quality of life, and acceptance of the disease, while the FCIS 2 subscale and FCIS 3 subscale assess the patient’s beliefs regarding the possible impact on the course of illness and the impact of the disease on the patient’s attitudes, respectively.

All participants signed an informed consent prior to the inclusion. This study received the approval of the Ethics Committee of The Nicolaus Copernicus University in Torun, Collegium Medicum in Bydgoszcz (KB 414/2021), and was conducted according to the Declaration of Helsinki and Good Clinical Practice principles.

The following endpoints were assessed after the 6-week rehabilitation program:

- Change in body composition according to gender;
- Change in muscle strength according to gender;
- Change in perceived fatigue assessed with the MFIS according to gender;
- Change in perceived dyspnea assessed with the mMRC dyspnea scale and the Borg scale according to gender;
- Change in exercise tolerance assessed with the 6MWT and the 30 CST according to gender;
- Change in physical fitness assessed with the SPPB test according to gender;
- Change in the functioning in chronic illness assessed with FCIS scale according to gender.

### Statistical Analysis

The statistical analysis was carried out using the Statistica 13.0 package (TIBCO Software Inc., Palo Alto, CA, USA). Continuous variables were presented as means with standard deviations. The Shapiro–Wilk test demonstrated a non-normal distribution of the investigated continuous variables. Therefore, non-parametric tests were used for statistical analysis. For comparison of continuous variables before and after post-COVID-19 rehabilitation, a parametric Wilcoxon signed rank test was used. Comparisons between men and women were performed with the Mann–Whitney unpaired rank sum test. The comparison of the gain (delta) of means between males and females was also performed. Results were considered significant at *p* < 0.05.

## 3. Results

A total of 90 patients (mean age 61.65 ± 5.39 years) with PCS were included in the final analysis, of which 41 were men (mean age 62.73 ± 5.24 years) and 49 were women (mean age 60.5 ± 5.54 years). The majority of patients were right-handed (*n* = 75, 83%), 11 patients were left-handed (12%), and 4 patients declared themselves as bimanual (4%). A total of 37 patients were hospitalized due to COVID-19, of whom 23 were males and 14 were females (56.1% vs. 28.6%, *p* = 0.01). Males more frequently had diabetes mellitus (24.4% vs. 8.2%, *p* = 0.03) and underwent myocardial infarction (19.5% vs. 4.1%, *p* = 0.047) and were less likely to have thyroid disorders (7.3% vs. 24.5%, *p* = 0.03). Detailed information regarding the baseline characteristics of the studied group is presented in Table 1.

Females had a lower waist circumference, decreased abdominal fat mass, increased skeletal muscle mass, lower hydration level, and improved phase angle after finishing the rehabilitation program. Among males, only a decreased waist circumference, hydration level, and increased phase angle were observed (Table 2).

In the female group, the rehabilitation program was associated with increased muscle mass in the left arm and non-dominant arm. In the male group, an increase in the right-hand and dominant-hand grip strength was observed, but not in the muscle mass (Table 3).

An improvement in the patients’ capacity and reduction in dyspnea assessed with functional scales (Borg’s scale, MFIS scale, and mMRC scale) were observed after the rehabilitation program. The mean walking distance in 6MWT increased and patients’ scores were higher in the 30 CST and SPPB test, both in the male and female groups (Table 4).

Patients had higher scores in all parts of the FCIS scale after the rehabilitation program and the same trend was observed regardless of gender (Table 5). Males had higher absolute mean values in all components of the FCIS scale both before and after the rehabilitation in comparison to females.

The changes in the mean values (delta) before and after the rehabilitation in males and females are presented in the appropriate tables (Table 2, Table 3, Table 4 and Table 5). In all analyzed parameters, the deltas did not differ between the genders (*p* > 0.05).

## 4. Discussion

The COVID-19 pandemic led to almost 7 million deaths worldwide [14], and many patients were affected by PCS. Rehabilitation has been shown to be an effective tool to minimize the long-term effects of COVID-19 [1,2]. According to Jimeno-Almazán et al., physical exercise is associated with short-, medium-, and long-term health benefits [15]. Physical activity was proven to be beneficial in various chronic diseases and to improve patients’ emotional and psychological state [15,16,17,18,19]. Physical inactivity may be associated with a potentially higher risk of severe consequences of COVID-19 [20]. According to Ceban et al., one-third of patients experienced chronic fatigue after COVID-19, which lasted much longer and even escalated in selected patients, contrary to other symptoms that usually ended spontaneously [4]. Previous research has shown that fatigue in patients with PCS was the longest-lasting symptom and affected 97.7% of individuals [21]. Fatigue related to COVID-19 infection occurred less frequently in the pediatric population while it was more common in females, elderly, and obese patients [4,21].

In our research, patients underwent the program of personalized PCS rehabilitation, which along with the multi-aspect assessment regarding physical capacity, anthropometric parameters, and functioning in chronic disease made the presented results uniquely valuable. The presented analysis addresses the need for the comprehensive assessment of differences in the effectiveness of PCS rehabilitation according to gender, because the available scientific evidence in this field up to date is scarce. The improvement in physical capacity, as expected, was observed both in men and women in all analyzed scales. Interestingly, in the female group, the muscle mass of the non-dominant arm increased without a significant improvement in the grip strength, while the opposite was seen in men—an improvement in the dominant-hand grip strength without a significant increase in the muscle mass. This might be associated with the initial differences in body composition between the groups, especially regarding the percentage of fat tissue. Hormonal factors might also play an important role [22]. In a study conducted on long-distance runners, the differences between men and women were mainly noted in the percentage of fat tissue and less regarding the cardiopulmonary capacity [23]. In the metanalysis assessing the effects of resistance training, the increase in upper-body strength was greater in the female group with a similar effect on the muscle hypertrophy and lower-body strength, which the authors mainly associated with the adaptation of the neurological system [22]. Therefore, the type of training may also have an impact on the observed effects of the rehabilitation.

Liu et al. showed an improvement in respiratory functions and walking distance in 6MWT in post-COVID-19 patients who underwent a 6-week respiratory rehabilitation program [24]. Similar results regarding 6MWT were observed by Ibrahim et al., who investigated the impact of aerobic, low-, and moderate-intensity exercises performed in 40 min sessions for 10 weeks in post-COVID-19 patients. This form of physical activity improved the functioning of the immune system, quality of life, as well as mental conditions, and increased the physical capacity of the patients [25]. Takekawa et al. reported that post-COVID-19 rehabilitation aimed at improving the gait pattern along with strengthening the trunk muscles increased the volume of the lumbar muscle [26].

Previous research showed a negative impact of PCS on body composition, muscle function, and quality of life [27]. In patients with PCS, rehabilitation has improved body composition, not only by increasing skeletal muscle mass but also by reducing fat mass and waist circumference [27,28]. Both the reduction in fat tissue and waist circumference are beneficial in terms of the risk of various chronic diseases, especially affecting the cardiovascular system [29,30,31]. In the PCR-SIRIO 8 study, the reduction in the abdominal fat volume and related reduction in the waist circumference were observed and were especially pronounced in the female group. 

One of the analyzed parameters was a phase angle measured before and after the rehabilitation program. The phase angle is used as an indicator of cellular health, integrity, and hydration [11]. A value above 6° is considered normal, and a value below 4.8° is an independent predictor of mortality in intensive care units [11,32,33]. It has been shown that the phase angle is inversely correlated with the length of in-hospital stay, and its low value was associated with a higher risk of mortality in patients hospitalized due to COVID-19 [32]. Our results revealed a significant improvement in the phase angle in the studied group regardless of gender; however, it should be underlined that the mean value of the phase angle was low (<6°) both before and after the rehabilitation.

Patients who underwent the program of personalized rehabilitation were also educated on physical activity, healthy diet, and ways to deal with post-COVID-19 complications. Therapeutic education along with physical activity are fundamental for prophylaxis and the treatment of various chronic diseases [1]. Therapeutic education enables patients to better understand the necessity of adequate diagnosis and treatment, helps to accept the proposed procedures, and increases the general effectiveness of the treatment [34]. The observed beneficial impact of the rehabilitation on the physical capacity along with the improvement in the anthropometric parameters of PCS patients translated into better functioning of men and women in this chronic illness.

The presented analysis stands out from previous publications on rehabilitation in PCS patients by showing detailed gender-related differences in anthropometric parameters and functioning after a rehabilitation program. The rehabilitation led to the improvement in functioning, physical capacity, and body composition regardless of gender. Although there were no differences in deltas between men and women, gender-related differences regarding the effect of the rehabilitation program could be seen, particularly on body composition and muscle strength. The reduction in waist circumference was observed both in males and females, but only in the latter group was the increase in skeletal muscle mass and reduction in fat tissue observed. This might be related to constitutional differences in body composition between genders. Males have more lean mass, less fat mass, and a lower fat-to-muscle mass ratio [35,36]. A crucial observation is that the rehabilitation both in males and females led to an improvement in fatigue, dyspnea, general physical capacity, and balance. Therefore, it is an effective tool to reduce the symptoms of PCS regardless of gender. Nevertheless, further research to evaluate the optimal rehabilitation strategies for reducing the negative consequences of COVID-19 is needed.

A major study limitation was the relatively low number of patients included in the analysis. Furthermore, no information regarding potential and additional physical activities as well as patients’ diet was available. Both these factors might influence the body composition, especially in terms of fat mass. The presented study is a post-hoc analysis of the gender-related differences in the population of the PCR-SIRIO 8 study, which was initially designed to investigate the improvement in patients’ functioning after a multidisciplinary, personalized rehabilitation program in PCS patients. Only patients who received general practitioner referral for PCS rehabilitation were included in this study. This could have potentially limited the recruitment; however, the initial assessment by the general practitioner was beneficial in terms of the confirmation of COVID-19 infection and screening for eligibility for PCS rehabilitation.

## 5. Conclusions

Rehabilitation in patients with PCS improved their functioning and physical capacity. Completion of the program led to an improvement in the proportion between fat and muscle mass, hydration, and phase angle both in the female and male subgroups. Rehabilitation should be applied in all patients with PCS regardless of gender-related differences in outcomes.

## Figures and Tables

**Table 1:** Baseline characteristic of the studied population. (BMI—body mass index; COPD—chronic obstructive pulmonary disease; COVID-19—coronavirus disease 2019; SD—standard deviation).

Analyzed Parameter	Studied Group (*n* = 90)	Females (*n* = 49)	Males (*n* = 41)	*p*-Value
Mean age (SD), [years]	61.65 ± 5.39	60.5 ± 5.54	62.73 ± 5.24	0.49
History of >1 COVID-19 infection, *n* (%)	2 (2.2)	1 (2.0)	1 (2.4)	0.56
Hospitalization due to COVID-19, *n* (%)	37 (41.1)	14 (28.6)	23 (56.1)	0.01
Right-handedness, *n* (%)	75 (83.3)	42 (85.7)	33 (80.5)	0.51
Employment, *n* (%)	44 (48.9)	24 (49.0)	20 (48.8)	0.99
Current smoker, *n* (%)	11 (12.2)	6 (12.2)	5 (12.2)	0.99
BMI ≥ 30.0 kg/m^2^, *n* (%)	28 (31.1)	13 (26.5)	15 (36.6)	0.31
Hypertension, *n* (%)	38 (42.2)	17 (34.7)	21 (51.2)	0.11
Diabetes mellitus, *n* (%)	14 (15.5)	4 (8.2)	10 (24.4)	0.03
Osteoarthritis, *n* (%)	23 (25.6)	16 (32.7)	7 (17.1)	0.09
History of myocardial infarction, *n* (%)	10 (11.1)	2 (4.1)	8 (19.5)	0.047
Bronchial asthma, *n* (%)	4 (4.4)	2 (4.1)	2 (4.9)	0.74
Bronchiectasis, *n* (%)	1 (1.1)	1 (2.0)	0 (0.0)	0.93
COPD, *n* (%)	12 (13.3)	7 (14.3)	5 (12.2)	0.77
Thyroid disorders, *n* (%)	15 (16.7)	12 (24.5)	3 (7.3)	0.03

**Table 2:** Analysis of the body composition in the studied group before and after the 6-week rehabilitation program. (BMI—body mass index; SD—standard deviation).

Analyzed Parameters	Studied Group (*n* = 90)			Females (*n* = 49)				Males (*n* = 41)				
	Before	After	*p*-Value	Before	After	Delta of the Mean ± SD	*p*-Value	Before	After	Delta of the Mean ± SD	*p*-Value	*p*-Value for Deltas
	Mean ± SD	Mean ± SD		Mean ± SD	Mean ± SD			Mean ± SD	Mean ± SD			
Total body mass, [kg]	79.85 ± 15.85	80.11 ± 15.82	0.68	74.17 ± 13.99	74.27 ± 14.11	0.10 ± 2.01	0.92	86.64 ± 15.42	87.10 ± 15.05	0.46 ± 3.06	0.65	0.83
BMI, [kg/m^2^]	27.91 ± 4.61	27.90 ± 4.57	0.51	27.86 ± 5.01	27.82 ± 5.01	−0.04 ± 0.76	0.42	27.96 ± 4.14	27.99 ± 4.04	0.03 ± 0.88	0.86	0.73
Waist circumference, [m]	0.96 ± 0.14	0.95 ± 0.14	0.001	0.92 ± 0.13	0.91 ± 0.13	−0.01 ± 0.04	0.03	1.02 ± 0.13	1.01 ± 0.12	−0.01 ± 0.02	0.01	0.57
Abdominal fat volume, [L]	3.03 ± 1.59	2.85 ± 1.56	0.001	2.31 ± 0.93	2.09 ± 1.06	−0.21 ± 0.53	0.02	3.89 ± 1.6	3.76 ± 1.58	−0.13 ± 0.64	0.21	0.80
Skeletal muscle mass, [kg]	24.11 ± 6.61	24.37 ± 6.61	0.01	19.96 ± 4.3	20.25 ± 4.28	0.29 ± 0.94	0.01	29.08 ± 5.36	29.31 ± 5.41	0.23 ± 1.20	0.25	0.35
Fat-free mass, [kg]	51.89 ± 11.56	52.14 ± 11.64	0.24	44.67 ± 7.61	44.81 ± 7.57	0.14 ± 1.85	0.59	60.51 ± 9.38	60.90 ± 9.38	0.39 ± 2.19	0.23	0.61
Rate of fat-free mass, [%]	64.85 ± 9.23	65.63 ± 8.86	0.39	61.20 ± 8.16	61.10 ± 8.14	−0.11 ± 2.45	0.60	69.21 ± 8.59	71.04 ± 6.34	1.82 ± 8.34	0.44	0.78
Fat mass, [kg]	27.95 ± 9.52	27.98 ± 9.53	0.83	29.48 ± 10.47	29.46 ± 10.29	−0.03 ± 1.66	0.81	26.13 ± 7.98	26.21 ± 8.32	0.08 ± 2.78	0.94	0.93
Rate of fat mass, [%]	34.81 ± 8.49	34.60 ± 8.46	0.44	39.00 ± 8.26	38.90 ± 8.14	−0.10 ± 1.84	0.53	29.81 ± 5.6	29.45 ± 5.46	−0.36 ± 2.48	0.62	0.98
Total body water, [L]	38.66 ± 8.62	38.76 ± 8.41	0.37	33.43 ± 5.54	33.60 ± 5.55	0.17 ± 1.34	0.44	44.90 ± 7.42	44.93 ± 6.99	0.03 ± 2.16	0.58	0.84
Rate of total body water, [%]	48.16 ± 5.78	48.43 ± 5.86	0.24	45.33 ± 5.59	45.67 ± 5.85	0.33 ± 1.84	0.29	51.53 ± 3.94	51.72 ± 3.85	0.19 ± 1.68	0.65	0.81
Extracellular water, [L]	17.43 ± 3.18	17.39 ± 3.24	0.22	15.72 ± 2.31	15.66 ± 2.4	−0.06 ± 0.78	0.53	19.48 ± 2.85	19.47 ± 2.91	−0.01 ± 0.78	0.23	0.94
Rate of extracellular water, [%]	21.93 ± 2.52	23.65 ± 18.0	0.37	21.44 ± 2.8	24.65 ± 24.41	3.21 ± 24.69	0.54	22.50 ± 2.04	22.45 ± 1.88	−0.05 ± 0.86	0.55	0.89
Hydration, [%]	83.54 ± 10.24	82.72 ± 9.25	0.001	88.41 ± 10.29	87.42 ± 8.52	−0.99 ± 5.71	0.004	77.72 ± 6.51	77.10 ± 6.63	−0.62 ± 1.72	0.01	0.09
Phase angle, [◦]	4.89 ± 0.74	5.01 ± 9.25	0.001	4.65 ± 0.64	4.77 ± 0.62	0.12 ± 0.22	0.001	5.17 ± 0.76	5.29 ± 0.72	0.12 ± 0.25	0.006	0.73

**Table 3:** Analysis of the muscle mass of the extremities and the grip strength before and after the 6-week rehabilitation program. (SD—standard deviation).

Analyzed Parameters	Studied Group (*n* = 90)			Females (*n* = 49)				Males (*n* = 41)				
	Before	After	*p*-Value	Before	After	Delta of the Mean ± SD	*p*-Value	Before	After	Delta of the Mean ± SD	*p*-Value	*p*-Value for Delta
	Mean ± SD	Mean ± SD		Mean ± SD	Mean ± SD			Mean ± SD	Mean ± SD			
Muscle mass of the right arm, [kg]	1.49 ± 1.35	1.50 ± 1.35	0.52	1.16 ± 1.12	1.16 ± 1.13	0.00 ± 0.00	0.88	1.90 ± 1.91	1.91 ± 1.99	0.01 ± 0.01	0.49	0.66
Muscle mass of the left arm, [kg]	1.43 ± 1.31	1.44 ± 1.34	0.02	1.09 ± 1.07	1.11 ± 1.09	0.02 ± 0.02	0.01	1.83 ± 1.85	1.84 ± 1.96	0.01 ± 0.00	0.44	0.48
Muscle mass of the right leg, [kg]	5.15 ± 5.09	5.18 ± 5.04	0.21	4.49 ± 4.38	4.51 ± 4.43	0.02 ± 0.02	0.38	5.94 ± 5.88	5.98 ± 6.10	0.04 ± 0.05	0.39	0.94
Muscle mass of the left leg, [kg]	5.15 ± 5.04	5.20 ± 5.05	0.05	4.49 ± 4.37	4.54 ± 4.39	0.06 ± 0.06	0.08	5.93 ± 5.92	5.98 ± 6.11	0.05 ± 0.03	0.40	0.47
Muscle mass of the dominant arm, [kg]	1.50 ± 1.32	1.49 ± 1.30	0.90	1.16 ± 1.12	1.16 ± 1.13	0.00 ± 0.00	0.90	1.91 ± 1.96	1.90 ± 1.98	−0.00 ± 0.01	0.10	0.97
Muscle mass of the non-dominant arm, [kg]	1.43 ± 1.30	1.45 ± 1.32	0.02	1.10 ± 1.07	1.12 ± 1.14	0.02 ± 0.02	0.02	1.84 ± 1.93	1.85 ± 1.96	0.01 ± 0.00	0.28	0.59
Right-hand grip strength, [kg]	12.15 ± 12.00	12.66 ± 12.00	0.001	10.96 ± 10.00	11.02 ± 11.00	0.02 ± 0.00	0.28	13.59 ± 12.00	14.57 ± 14.00	0.98 ± 0.00	0.0003	0.13
Left-hand grip strength, [kg]	12.16 ± 12.00	12.44 ± 12.50	0.03	10.57 ± 11.00	10.73 ± 11.00	0.17 ± 0.00	0.13	14.09 ± 13.00	14.45 ± 14.00	0.36 ± 0.00	0.08	0.74
Dominant-hand grip strength, [kg]	12.30 ± 12.00	12.60 ± 12.00	0.01	11.06 ± 11.00	10.81 ± 11.00	−0.06 ± 0.00	0.67	13.83 ± 13.00	14.81 ± 14.00	0.98 ± 0.00	0.0002	0.08
Non-dominant-hand grip strength, [kg]	12.09 ± 12.00	12.10 ± 12.00	0.21	10.52 ± 10.00	10.33 ± 10.00	0.21 ± 0.00	0.46	14.02 ± 13.00	14.29 ± 14.00	0.26 ± 0.00	0.22	0.44

**Table 4:** Analysis of self-reported fatigue, dyspnea, general physical capacity, and balance before and after the 6-week rehabilitation program. (MFIS—Modified Fatigue Impact Scale; mMRC—modified Medical Research Council scale; SPPB—Short Physical Performance Battery test; 30 CST—30 s chair stand test; 6MWT—Six-Minute Walk Test).

Analyzed Parameter	Studied Group (*n* = 90)			Females (*n* = 49)				Males (*n* = 41)				
	Before	After	*p*-Value	Before	After	Delta of the Mean ± SD	*p*-Value	Before	After	Delta of the Mean ± SD	*p*-Value	*p*-Value for Delta
	Mean ± SD	Mean ± SD		Mean ± SD	Mean ± SD			Mean ± SD	Mean ± SD			
Borg’s scale, [pts]	3.65 ± 1.95	2.26 ± 1.44	<0.0001	3.92 ± 2.13	2.25 ± 1.41	−1.72 ± 2.11	<0.0001	3.34 ± 1.70	2.26 ± 1.50	−1.11 ± 2.22	0.002	0.36
MFIS scale, [pst]	37.37 ± 15.49	29.14 ± 14.23	<0.0001	41.19 ± 14.82	31.35 ± 14.51	−10.12 ± 12.42	<0.0001	32.77 ± 15.17	26.39 ± 13.54	−6.93 ± 10.77	0.001	0.41
30CST, [pts]	13.39 ± 4.32	17.10 ± 4.66	<0.0001	13.19 ± 3.78	16.63 ± 4.24	3.41 ± 2.16	<0.0001	13.63 ± 4.93	17.67 ± 5.12	4.12 ± 3.23	<0.0001	0.41
6MWT, [m]	327.80 ± 82.88	383.96 ± 74.59	<0.0001	318.33 ± 89.19	370.04 ± 71.36	47.82 ± 50.76	<0.0001	339.80 ± 73.43	401.28 ± 75.73	61.48 ± 43.75	<0.0001	0.18
SPPB scale, [pts]	13.68 ± 2.09	15.09 ± 1.32	<0.0001	13.43 ± 2.28	15.08 ± 1.21	1.53 ± 1.72	<0.0001	13.98 ± 1.79	15.10 ± 1.46	1.05 ± 1.34	<0.0001	0.20
mMRC scale, [pts]	2.21 ± 0.52	1.00 ± 0.89	<0.0001	2.28 ± 0.57	0.96 ± 0.89	−1.31 ± 0.93	<0.0001	2.11 ± 0.44	1.05 ± 0.89	−1.07 ± 0.85	<0.0001	0.15
Desaturation during 6MWT, [*n* (%)]	4 (4.4)	0 (0.0)	0.13	2 (4.1)	0 (0.0)	N/A	0.48	2 (4.9)	0 (0.0)	N/A	0.47	N/A

**Table 5:** Results of the Functioning in Chronic Illness Scale (FICS) before and after the 6-week rehabilitation program. (SD—standard deviation).

Analyzed Parameters	Studied Group (*n* = 90)			Females (*n* = 49)				Males (*n* = 41)				
	Before	After	*p*-Value	Before	After	Delta of the Mean ± SD	*p*-Value	Before	After	Delta of the Mean ± SD	*p*-Value	*p*-Value for Delta
	Mean ± SD	Mean ± SD		Mean ± SD	Mean ± SD			Mean ± SD	Mean ± SD			
FCIS 1 [pts]	25.62 ± 6.53	27.85 ± 5.65	<0.0001	24.81 ± 6.54	26.81 ± 5.72	2.00 ± 3.43	0.001	26.59 ± 6.46	29.14 ± 5.34	2.44 ± 3.78	0.001	0.34
FCIS 2 [pts]	28.74 ± 4.06	30.46 ± 4.57	<0.0001	28.08 ± 3.80	29.28 ± 4.38	1.21 ± 2.73	0.005	29.55 ± 4.27	31.91 ± 4.42	2.14 ± 3.71	0.001	0.13
FCIS 3 [pts]	31.71 ± 4.05	33.40 ± 3.80	<0.0001	31.34 ± 3.99	32.74 ± 3.95	1.40 ± 2.96	0.001	32.16 ± 4.13	34.21 ± 3.49	1.86 ± 3.31	0.003	0.31
FCIS total [pts]	86.07 ± 11.89	91.71 ± 11.51	<0.0001	84.23 ± 11.13	88.83 ± 11.84	4.60 ± 6.00	<0.0001	88.30 ± 12.50	95.26 ± 10.13	6.44 ± 9.24	<0.0001	0.15

## Data Availability

The raw data supporting the conclusions of this article will be made available by the authors on request.

## References

[1] Ostrowska M., Rzepka-Cholasińska A., Pietrzykowski Ł., Michalski P., Kosobucka-Ozdoba A., Jasiewicz M., Kasprzak M., Kryś J., Kubica A. (2023). Effects of multidisciplinary rehabilitation program in patients with long COVID-19: Post-COVID-19 rehabilitation (PCR SIRIO 8) study. J. Clin. Med..

[2] Kubica A., Michalski P., Pietrzykowski Ł., Rzepka-Cholasińska A., Kosobucka-Ozdoba A., Jasiewicz M., Laskowska E., Kryś J., Ostrowska M. (2022). Post-COVID-19 rehabilitation (PCR-SIRIO 8) study: A rationale and protocol of the study. Med. Res. J..

[3] Huang C., Huang L., Wang Y., Li X., Ren L., Gu X., Kang L., Guo L., Liu M., Zhou X. (2021). 6-month consequences of COVID-19 in patients discharged from hospital: A cohort study. Lancet.

[4] Ceban F., Ling S., Lui L.M.W., Lee Y., Gill H., Teopiz K.M., Rodrigues N.B., Subramaniapillai M., Vincenzo J.D.D., Cao B. (2022). Fatigue and cognitive impairment in Post-COVID-19 Syndrome: A systematic review and meta-analysis. Brain Behav. Immun..

[5] Kubica A., Michalski P., Kasprzak M., Podhajski P., Pietrzykowski Ł., Rzepka-Cholasińska A., Fabiszak T., Kryś J. (2021). Functioning of patients with post-COVID syndrome: Preliminary data. Med. Res. J..

[6] Rengo J.L., Khadanga S., Savage P.D., Ades P.A. (2020). Response to Exercise Training During Cardiac Rehabilitation Differs by Sex. J. Cardiopulm. Rehabil. Prev..

[7] Moro T., Marcolin G., Bianco A., Bolzetta F., Berton L., Sergi G., Paoli A. (2020). Effects of 6 Weeks of Traditional Resistance Training or High Intensity Interval Resistance Training on Body Composition, Aerobic Power and Strength in Healthy Young Subjects: A Randomized Parallel Trial. Int. J. Environ. Res. Public Health.

[8] Khadanga S., Savage P.D., Pecha A., Khadanga S., Savage P.D., Pecha A., Rengo J., Ades P.A. (2022). Optimizing Training Response for Women in Cardiac Rehabilitation: A Randomized Clinical Trial. JAMA Cardiol..

[9] Carter S.J., Baranauskas M.N., Raglin J.S., Pescosolido B.A., Perry B.L. (2022). Functional Status, Mood State, and Physical Activity Among Women with Post-Acute COVID-19 Syndrome. Int. J. Public Health.

[10] Papaemmanouil A., Bakaloudi D.R., Gkantali K., Papaemmanouil A., Bakaloudi D.R., Gkantali K., Kalopitas G., Metallidis S., Germanidis G., Chourdakis M. (2023). Phase Angle and Handgrip Strength as Predictors of Clinical Outcomes in Hospitalized COVID-19 Patients. Nutrients.

[11] Moonen H.P., Bos A.E., Hermans A.J., Stikkelman E., van Zanten F.J., van Zanten A.R. (2021). Bioelectric impedance body composition and phase angle in relation to 90-day adverse outcome in hospitalized COVID-19 ward and ICU patients: The prospective BIAC-19 study. Clin. Nutr. ESPEN.

[12] Cornejo-Pareja I., Vegas-Aguilar I.M., García-Almeida J.M., Diego Bellido-Guerrero D., Antonio Talluri A., Lukaski H., Tinahones F.J. (2021). Phase angle and standardized phase angle from bioelectrical impedance measurements as a prognostic factor for mortality at 90 days in patients with COVID-19: A longitudinal cohort study. Clin. Nutr..

[13] Michalski P., Kasprzak M., Kosobucka-Ozdoba A., Pietrzykowski Ł., Kieszkowska M., Bączkowska A., Kubica A. (2022). The impact of knowledge on the functioning of patients with coronary artery disease. Med. Res. J..

[14] Number of COVID-19 Cases Reported to WHO. https://covid19.who.int.

[15] Jimeno-Almazán A., Franco-López F., Buendía-Romero A., Martínez-Cava A., Sánchez-Agar J.A., Sánchez-Alcaraz Martínez B.J., Courel-Ibáñez J., Pallarés J.G. (2022). Rehabilitation for post-COVID-19 condition through a supervised exercise intervention: A randomized controlled trial. Scand. J. Med. Sci. Sports.

[16] Jimeno-Almazán A., Martínez-Cava A., Buendía-Romero A., Franco-López F., Sánchez-Agar J.A., Sánchez-Alcaraz B.J., Tufano J.J., Pallarés J.G., Courel-Ibáñez J. (2022). Relationship between the severity of persistent symptoms, physical fitness, and cardiopulmonary function in post-COVID-19 condition. A population-based analysis. Intern. Emerg. Med..

[17] Li M.L., Kor P.P.-K., Sui Y.F., Liu J.Y.-W. (2023). Health maintenance through home-based interventions for community-dwelling older people with sarcopenia during and after the COVID-19 pandemic: A systematic review and meta-analysis. Exp. Gerontol..

[18] Daynes E., Gerlis C., Chaplin E., Gardiner N., Singh S.J. (2021). Early experiences of rehabilitation for individuals post-COVID to improve fatigue, breathlessness exercise capacity and cognition—A cohort study. Chron. Respir. Dis..

[19] Nigro E., Polito R., Alfieri A., Mancini A., Imprerlini E., Elce A., Krustrup P., Orru S., Buono P. (2020). Molecular mechanisms involved in the positive effects of physical activity on coping with COVID-19. Eur. J. Appl. Physiol..

[20] Chen X., Hong X., Gao W., Luo S., Cai J., Liu G., Huang Y. (2022). Causal relationship between physical activity, leisure sedentary behaviors and COVID-19 risk: A Mendelian randomization study. Transl. Med..

[21] Sudre C.H., Murray B., Varsavsky T., Graham M.S., Penfold R.S., Bowyer R.C., Pujol J.C., Klaser K., Antonelli M., Canas L.S. (2021). Attributes and predictors of long COVID. Nat. Med..

[22] Roberts B.M., Nuckols G., Krieger J.W. (2020). Sex Differences in Resistance Training: A Systematic Review and Meta-Analysis. J. Strength Cond. Res..

[23] Lewis D.A., Kamon E., Hodgson J.L. (1986). Physiological differences between genders. Implications for sports conditioning. Sports Med..

[24] Liu K., Zhang W., Yang Y. (2020). Respiratory rehabilitation in elderly patients with COVID-19: A randomized controlled study. Complement. Ther. Clin. Pract..

[25] Ibrahim A.A., Hussein H.M., Ali M.S., Kanwal R., Acar T., Shaik D.H., Alghamdi W., Althomal O.W. (2023). A randomized controlled trial examining the impact of low vs. moderate-intensity aerobic training in post-discharge COVID-19 older subjects. Eur. Rev. Med. Pharmacol. Sci..

[26] Takekawa T., Kashiwabara K., Yamada N., Watanabe S., Hama M., Hashimoto G., Abo M., Shinfuku K. (2022). Rehabilitation therapy for a severe case of coronavirus disease 2019: A case report. J. Med. Case Rep..

[27] González-Islas D., Sánchez-Moreno C., Orea-Tejeda A., Hernandez-Lopez S., Salgad-Fernandez F., Keirns-Davis C., Galicia-Amour S., Trejo-Mellando E., Gochicoa-Rangel L., Castorena-Maldonado A. (2022). Body composition and risk factors associated with sarcopenia in post-COVID patients after moderate or severe COVID-19 infections. BMC Pulm. Med..

[28] Yong S.J. (2021). Long COVID or post-COVID-19 syndrome: Putative pathophysiology, risk factors, and treatments. Infect. Dis..

[29] Halpin S.J., McIvor C., Whyatt G., Adams A., Harvey O., McLean L., Walshaw C., Kemp S., Corrado J., Singh R. (2021). Postdischarge symptoms and rehabilitation needs in survivors of COVID-19 infection: A cross-sectional evaluation. J. Med. Virol..

[30] Khalangot M., Sheichenko N., Gurianov V., Vlasenko V., Kurinna J., Samson O., Tronko M. (2022). Relationship between hyperglycemia, waist circumference, and the course of COVID-19: Mortality risk assessment. Exp. Biol. Med..

[31] Nalbandian A., Sehgal K., Gupta A., Madhavan M.V., McGroder C., Stevens J.S., Cook J.R., Nordvig A.S., Shalev D., Sehrawat T.S. (2021). Post-acute COVID-19 syndrome. Nat. Med..

[32] Moonen H.P.F.X., Van Zanten A.R.H. (2021). Bioelectric impedance analysis for body composition measurement and other potential clinical applications in critical illness. Curr. Opin. Crit. Care.

[33] Moonen H.P.F.X., van Zanten F.J.L., Driessen L., de Smet V., Slingerland-Boot R., Mensink M., Raymound A., van Zanten H. (2021). Association of bioelectric impedance analysis body composition and disease severity in COVID-19 hospital ward and ICU patients: The BIAC-19 study. Clin Nutr..

[34] Niksadat N., Rakhshanderou S., Negarandeh R., Ramezankhani A., Farahani A., Ghaffari M. (2022). Concordance of the cardiovascular patient education with the principles of Andragogy model. Arch. Public Health.

[35] Yu B., Sun Y., Du X., Zhang H., Chen C., Tan X., Yang Z., Lu Y., Wang N. (2023). Age-specific and sex-specific associations of visceral adipose tissue mass and fat-to-muscle mass ratio with risk of mortality. J. Cachexia Sarcopenia Muscle.

[36] Bredella M.A. (2017). Sex Differences in Body Composition. Adv. Exp. Med. Biol..

